# Relationship between cognitive behavioral variables and mental health status among university students: A meta-analysis

**DOI:** 10.1371/journal.pone.0223310

**Published:** 2019-09-27

**Authors:** Tomonari Irie, Kengo Yokomitsu, Yuji Sakano

**Affiliations:** 1 School of Education and Culture, Hokusho University, Hokkaido, Japan; 2 Graduate School of Psychological Science, Health Sciences University of Hokkaido, Hokkaido, Japan; 3 College of Comprehensive Psychology, Ritsumeikan University, Osaka, Japan; 4 School of Psychological Science, Health Sciences University of Hokkaido, Hokkaido, Japan; Universita degli studi di Padova (Padua University), ITALY

## Abstract

Cognitive behavioral therapy is an effective treatment for improving mental health problems among university students. However, intervention components have different effects on mental health problems. This paper is a meta-analysis of the data concerning the relationship between cognitive behavioral variables and mental health status among university students. A total of five electronic databases were reviewed, and 876 articles met the initial selection criteria. Reviewers applied standardized coding schemes to extract the correlational relationship between cognitive behavioral variables and mental health status. A total of 55 articles were included in the meta-analysis. Correlations were found for three cognitive behavioral variables (attention, thought, and behavior) across nine mental health domains (negative affect, positive affect, happiness, social function, stress response, psychological symptom, quality of life, well-being, and general health). Across each cognitive behavioral process and all mental health domains, the estimated mean correlation was medium (*r* = .32 - .46), and varied by the domain of mental health.

## Introduction

Mental health problems among university students is an important issue. Auerbach et al. analyzed data on mental health problems of university students in each country using the World Mental Health Surveys [1]. The results indicated that university students suffering from psychiatric disorders were reluctant to attend university and were unable to receive appropriate treatment. Steptoe et al. investigated the extent of depressive symptoms in 17,348 university students between the ages of 17 and 30 in 23 countries [2]. They found that the prevalence of severe depressive symptoms was 38% in university students from East Asia (e.g., Japan, Korea), 13.9% for men and 17.1% for women from Western countries. Therefore, establishing and managing a support system in universities that focuses on mental health problems has become an important issue.

Mental health problems among university students have negative influences on academic performance and social function. Richardson et al. conducted a meta-analysis of 217 studies that examined the relationship between mental health status and academic performance [3]. This analysis revealed that the intensity of general stress has a negative impact on academic performance. Weissman et al. investigated the influence of depressive and anxiety symptoms on daily life in adolescence and on social life throughout adulthood [4]. This study showed that depressive and anxiety symptoms in adolescence influenced their job turnover rate and the likelihood that they will remain unmarried.

The WHO definition of health emphasizes not only the absence of illness but also positive aspects such as social functioning and well-being [5]. Keyes classified the positive aspects of mental health in terms of hedonia and positive functioning [6]. Specifically, hedonia includes “experiencing positive affect” and “avowing happiness or life satisfaction”; in addition, positive functioning includes “social acceptance,” “social actualization,” “social contribution,” “social coherence,” “social integration,” “personal growth,” “purpose in life,” “autonomy,” “environmental mastery,” “self-acceptance,” and “positive relationships with others.” Furthermore, showing “personal growth,” “purpose in life,” “autonomy,” “environmental mastery,” “self-acceptance,” and “positive relationships with others” are components of well-being [7]. Therefore, in order to measure mental health, it is necessary to assess not only the general health condition including psychological symptoms or stress responses related to mental illness indicated by WHO, but also affective state, happiness, social functioning, and well-being.

Cognitive behavior therapy (CBT) is widely applied as a psychological approach to promote good mental health in university students. Charlesworth et al. examined the effect of relaxation training on state and trait anxiety in 18 college students [8]. The results indicated that relaxation training reduces trait anxiety. Perna et al. (1998) examined the effect of cognitive behavioral stress management program on the mood states in 34 university students [9]. The results showed that cognitive behavioral stress management program reduces dysphoric mood states. Rosenzweig et al. examined the effect of mindfulness training on mood states for 302 university students in their sophomore year [10]. They found that mindfulness training reduces dysphoric mood states. Levin et al. examined the effect of web-based acceptance and commitment therapy on the academic concerns and well-being of 79 college students [11]. The results indicated that acceptance and commitment therapy improved concerns about academic learning and social well-being.

CBT is an effective approach to improve mental health in university students. CBT includes many therapeutic components and outcome measures. Harvey et al. pointed out that there are five cognitive behavioral variables that can be applied to cognitive behavioral therapy (i.e., attention, memory, reasoning, thought, and behavior [12]). Attention includes variables such as selective attention and mindfulness. Memory includes variables such as overgeneral memory and memory distrust. Reasoning includes variables such as interpretation and attribution. Thought includes variables such as rumination and belief. Behavior includes variables such as avoidance and coping. These five variables are not disorder specific, and are applied in the transdiagnostic approach [12]. Conley et al. conducted a systematic review on the effects of psychological interventions for promoting mental health in university students [13]. The results indicated that mindfulness training is more effective than CBT, relaxation training, and meditation. Furthermore, CBT was found to be more effective than relaxation training and meditation.

Each component of psychological approaches to mental health problems of university students has a different effect. The outcome measures of cognitive behavioral variables that affect the mental health problems of university students have not been verified. Less than one in five adolescents who are in need of treatment receive appropriate psychological interventions [14]. To improve access to effective psychological interventions, it may be useful to develop a brief intervention [15]. In this way, the student counseling center at universities can offer psychological interventions during semester term [16,17]. Identifying cognitive behavioral variables that strongly influences mental health status is important for developing an effective protocol. In the present study, we aim to conduct an analysis to identify cognitive behavioral variables that influence mental health status in university students.

## Method

### Definition of terms

First, we defined cognitive behavioral variables according to Harvey et al’s definition [12]. The definition is as follows: (1) attention, (2) memory, (3) reasoning, (4) thought, and (5) behavior. Second, we defined mental health according to WHO’s definition of health and Keyes’s definition of positive aspects of mental health [5,6]. The definition is as follows: (1) negative affect, (2) positive affect, (3) happiness, (4) social function, (5) stress response, (6) psychological symptom, (7) quality of life (QOL), (8) well-being, and (9) general health.

### Search strategy

We identified relevant articles in multiple electronic databases (PsycINFO, PubMed, and CENTRAL). In addition, we used the SIGLE and PsyEXTRA databases to search grey literature. The search included articles published in English from the earliest date available to June 11, 2019 in each database. The selected search terms were “universities,” “college,” “undergraduate,” “mental processes,” “adaptation, psychological,” “attitude,” “attention,” “psychology,” and “mental health.” After the database search, we also searched the reference sections of the articles for additional sources. Additionally, the Thesaurus of Psychological Index Terms, created by the American Psychological Association, can be used as a type of thesaurus search in PsycINFO. Therefore, we utilized this additional tool when searching PsycINFO to obtain all possible references in addition to the above-mentioned terminologies.

### Inclusion and exclusion criteria

Studies included for meta-analysis met the following criteria: (1) written in English, (2) samples were specifically college or junior college students, (3) assessed a bivariate relationship between mental health status and cognitive behavioral variables, (4) reported an effect size, or a statistic that can be calculated, measuring the bivariate association between cognitive behavioral variables and mental health status, and (5) published in a peer-reviewed journal. It also included baseline data for intervention studies. Studies were excluded if their samples were psychiatric patients.

### Screening procedures

**Fig 1:**
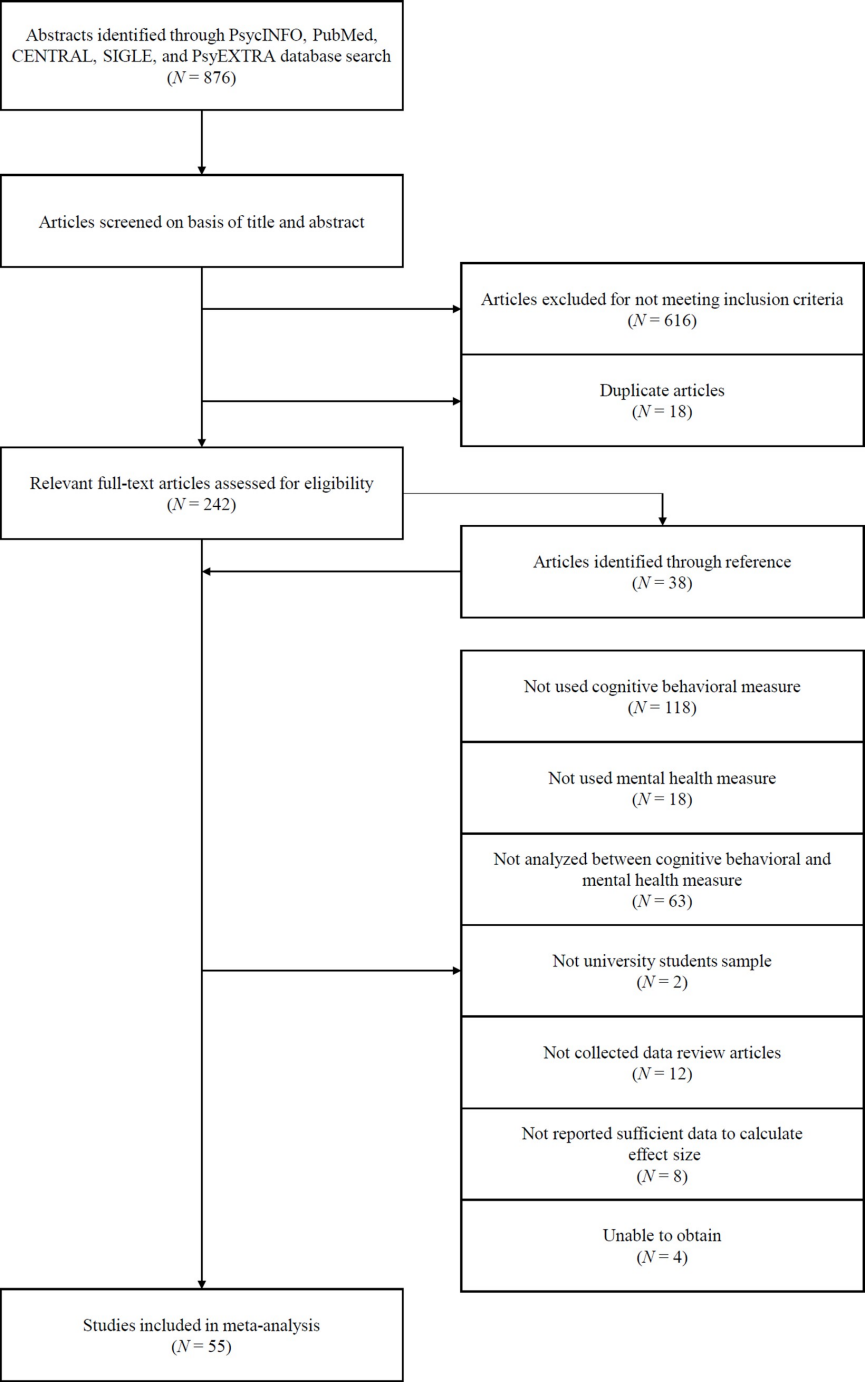
Flowchart of study selection.

Based on the inclusion criteria, two independent raters evaluated “include,” “exclude,” and “unsure” for each article. The value of Kappa indicates fair agreement (*κ* = .47) [18]. Of the 876 articles extracted using the electronic search, we rejected 616 articles for which both the raters evaluated “exclude.” This resulted in 260 articles, of which 27 articles received the same “include” evaluation by both raters, 66 articles received the same “unsure” evaluation by both raters, and 167 articles were evaluated as either “include” or “unsure” by either rater. There were 18 duplicates among the 260 articles. Therefore, we searched the reference sections of the 242 articles. As a result of the reference section search, we extracted 38 new articles. Two raters independently read the full texts of the 280 articles and judged whether they should be subject to meta-analysis. The inter-rater disagreement were resolved by discussion between the raters once they reached a consensus. Furthermore, as defined above, cognitive behavioral measures were categorized as attention, memory, reasoning, thought, or behavior, mental health measures were categorized as negative affect, positive affect, happiness, social function, stress response, psychological symptom, QOL, well-being, or general health. The inter-rater classification differences were resolved by discussions between raters based on the definition and a consensus was reached. A total of 55 articles were selected for the meta-analysis (Table 1). Fig 1 presents the procedure used to extract the articles.

**Table 1:** Characteristics of the included studies.

Study	*N*	Nationality	Cognitive behavioral variables	Mental health
Anderson & Arnoult (1989) [19]	159	USA	Thought	Negative affect, Psychological symptom
Berking et al. (2012) [20]	151	Germany	Attention, Thought, Behavior	Psychological symptom
Bettis et al. (2017) [21]	62	USA	Behavior	Stress response, Psychological Symptom
Birks et al. (2009) [22]	289	England	Behavior	Stress response
Bodenlos et al. (2015) [23]	310	USA	Attention	Social function, Stress response
Bowlin & Baer (2012) [24]	280	USA	Attention	Negative affect, Stress response, Well-being
Brittian et al. (2015) [25]	2315	USA	Thought	Psychological symptom
Calogero & Pina (2011) [26]	225	USA	Thought	Negative affect, Psychological symptom
Chen et al. (2014) [27]	113	USA	Thought	Psychological symptom
Coffey et al. (2010) [28]	413	USA	Attention, Thought, Behavior	Psychological symptom
Costa et al. (2013) [29]	1078	Spain, Mexico, Portugal,Brazil	Attention, Thought, Behavior	General health, Happiness
Deng et al. (2011) [30]	263	China	Attention	Negative affect, Positive affect, QOL
Disch et al. (2000) [31]	467	USA	Attention	Social function, Happiness
Flett et al. (2016) [32]	214	Canada	Attention, Thought	Negative affect
Gilbert & Christopher (2009) [33]	268	USA	Attention, Thought	Psychological symptom
Griva & Anagnostopoulos (2010) [34]	268	Greece	Behavior	Psychological symptom
Hintz et al. (2015) [35]	223	USA	Thought	Negative affect, Stress response
Hipwell (2005) [36]	183	Scotland	Behavior	Psychological symptom
Hovey & Seligman (2007) [37]	190	USA	Behavior	Psychological symptom
Iwasaki (2003) [38]	85	Canada	Behavior	Well-being, General health
Jayalakshmi & Magdalin (2015) [39]	125	India	Behavior	Well-being
Khan et al. (2016) [40]	207	Pakistan	Behavior	Psychological symptom
Kim et al. (2015) [41]	107	USA	Behavior	Well-being
Kneeland & Dovidio (2019) [42]	97	New Zealand	Thought	Stress response, Psychological Symptom
Koesten et al. (2009) [43]	395	USA	Behavior	General health
Kraemer et al. (2016) [44]	452	USA	Attention, Thought	Negative affect
Krafft et al. (2019) [45]	339	USA	Thought	Stress response
Lihua et al (2017) [46]	330	China	Behavior	Psychological symptom
Luo & Wang (2009) [47]	284	China	Behavior	Psychological symptom
Mahmoud et al. (2012) [48]	508	USA	Behavior	Negative affect, Stress response
Marino et al. (2016) [49]	795	Italy	Attention	Happiness
Masuda & Tully (2012) [50]	494	USA	Attention, Behavior	Psychological symptom, General health
Masuda & Wendell (2010) [51]	91	USA	Attention, Thought	Stress response, General health
Masuda et al. (2009) [52]	301	USA	Attention, Behavior	Stress response, General health
Masuda et al. (2010) [53]	375	USA	Thought, Behavior	Stress response, General health
Mayorga et al. (2018) [54]	448	USA	Behavior	Negative affect, Stress response, Psychological symptom
Moeller & Seehuus (2019) [55]	2054	USA	Behavior	Psychological symptom
Montes-Berges & Augusto (2007) [56]	119	Spain	Attention, Behavior	Psychological symptom
de Oliveira et al. (2015) [57]	184	Brazil	Thought	Psychological symptom
Ranjbar et al. (2013) [58]	369	Iran	Behavior	Social function, Psychological symptom, General health
Sanchez et al. (2018a) [59]	308	USA	Behavior	Psychological symptom
Sanchez et al. (2018b) [60]	211	USA	Behavior	Positive affect
Sasaki & Yamasaki (2005) [61]	292	Japan	Behavior	Social function, Psychological symptom
Shapiro et al. (2011) [62]	32	USA	Attention	Stress response, Well-being
Su & Chen (2015) [63]	110	Taiwan	Thought, Behavior	Psychological symptom
Thanoi & Klainin-Yobas (2015) [64]	747	Thailand	Thought	Negative affect, Stress response
Tucker et al. (2016) [65]	123	USA	Thought	Psychological symptom
Vand et al. (2014) [66]	400	Iran	Attention, Thought	Negative affect, Psychological symptom
Wang et al. (2016) [67]	262	Taiwan	Behavior	Negative affect, Positive affect, Social function
Wang et al. (2017) [68]	533	China	Attention	Psychological symptom
Wong (2010) [69]	398	Singapore	Thought	Happiness, Psychological symptom
Wong et al. (2014) [70]	160	USA	Thought	Psychological symptom
Woodruff et al. (2013) [71]	147	USA	Attention, Behavior	Negative affect, Positive affect, Happiness, Psychological symptom, QOL
Zawadzki et al. (2018) [72]	491	USA	Thought	Psychological symptom, General health
Zhou et al. (2013) [73]	418	China	Attention	Psychological symptom

### Meta-analytic procedures

This study targeted investigations reporting on the bivariate relationship between cognitive behavioral variables and mental health status. Therefore, multivariable measures of association, such as regression coefficients, were excluded because they are not directly comparable to measures of bivariate association [74]. A meta-analysis was conducted for each combination of cognitive behavioral process and mental health status. When multiple outcomes were used in the study, the effect sizes were extracted for each combination of classifications if the combination of classifications was different (e.g., combination of automatic thoughts and positive affect [thought and positive affect], and combination of automatic thoughts and depressive symptoms [thought and psychological symptoms]). When the effect size was reported in the same combination, it was integrated into the research (e.g., combination of automatic thoughts and depressive symptoms [thought and psychological symptoms] and combination of automatic thoughts and anxiety symptoms [thought and psychological symptoms]). To integrate the effect size, we used Fisher’s z scale weighted for sample size. Cohen’s standard definition of small (.10), medium (.30), and large (.50) effect sizes were used to interpret the effect size findings [75]. In a meta-analysis, clinical and statistical heterogeneity are inevitable because subjects and areas differ depending on the study [76]. Therefore, we used the random effect model to calculate the effect size. Furthermore, we calculated *I*^*2*^ [76], and the statistical heterogeneity of the research included in the meta-analysis was confirmed. If we detected a large heterogeneity, then we conducted a subgroup analysis based on the classification of mental health status (i.e., positive affect, negative affect etc.). To confirm publication bias, we examined the symmetry of the funnel plot using a linear regression test [77] and the trim and fill method [78]. For all analyses, we used the R version 3.4.1 [79]. We used the metafor package [80] to integrate effect size and examine the symmetry of the funnel plot.

## Results

### Characteristics of included studies

We extracted three categories of cognitive behavioral variables, “attention,” “thought,” and “behavior”, and all categories of mental health based on reading the full-text. The variables of “memory” and “reasoning” were not extracted. Table 1 presents the characteristics of the included studies. Furthermore, Table 2 presents the results of the classification, and Table 3 presents the scales used in each classification.

**Table 2:** Classification of the included studies.

Category	Studies	*N*
**Cognitive behavioral variables**		
Attention	20	36
Thought	23	33
Behavior	29	48
**Mental health**		
Negative affect	13	17
Positive affect	4	5
Happiness	4	8
Social function	5	5
Stress response	14	17
Psychological symptoms	32	41
Quality of life	2	3
Well-being	5	6
General Health	9	15

**Table 3:** Measures used in each classification.

Measure	Psychometrics^a^		Studies
	*α*	*r*_xx_	
**Attention**			
Mindful Attention Awareness Scale	.80	.81	Deng et al. (2011) [30]
(Brown & Ryan, 2003) [81]			Flett et al. (2016) [32]
			Gilbert & Christopher (2009) [33]
			Masuda & Tully (2012) [50]
			Masuda & Wendell (2010) [51]
			Masuda et al. (2009) [52]
			Shapiro et al. (2011) [62]
			Wang et al. (2017) [68]
			Woodruff et al. (2013) [71]
Five Facet Mindfulness Questionnaire	.75 - .91	-	Bodenlos et al. (2015) [23]
(Baer et al., 2006) [82]			Bowlin & Baer (2012) [24]
			Coffey et al. (2010) [28]
			Woodruff et al. (2013) [71]
Trait Meta-Mood Scale (subscale; Attention, Clarity)	.86 - .90	-	Coffey et al. (2010) [28]
(Salovey et al., 1995) [83]			Costa et al. (2013) [29]
			Montes-Berges & Augusto (2007) [56]
Metacognition questionnaire	.72 - .89	.76 - .94	Marino et al. (2016) [49]
(Cartwright-Hatton & Wells, 1997) [84]			Vand et al. (2014) [66]
Emotion-Regulation Skills Questionnaire (subscale; Awareness)	.90	.75	Berking et al. (2012) [20]
(Berking & Znoj, 2008) [85]			
Cognitive and Affective Mindfulness Scale-Revised	.74 - .77	-	Kraemer et al. (2016) [44]
(Feldman et al., 2007) [86]			
Self-Compassion Scale (subscale; Mindfulness)	.81	.85	Zhou et al. (2013) [73]
(Neff, 2003) [87]			
Lerning Styles Inventory (subscale; Deep cognitive processing)	.82	.88	Disch et al. (2000) [31]
(Schmeck, 1983) [88]			
**Thought**			
Objectified Body Consciousness Scale (subscale; Surveillance)	.76 - .89	-	Calogero & Pina (2011) [26]
(McKinley & Hyde, 1996) [89]			
Dysfunctional Belief and Attitudes about Sleep Scale	.69	-	Vand et al. (2014) [66]
(Morin, 1993) [90]			
Perceived Control Over Stressful Events Scale (subscale; Present control)	.79 - .86	.48 - .59	Hintz et al. (2015) [35]
(Frazier et al 2011) [91]			
Response Styles Questionnaire (subscale; Ruminative)	-	.80	Flett et al. (2016) [32]
(Nolen-Hoeksema & Morrow, 1991) [92]			Su & Chen (2015) [63]
(Nolen-Hoeksema et al., 1994) [93]			Thanoi & Klainin-Yobas (2015) [64]
Intolerance of Uncertainty Scale	.94	.74	Kraemer et al. (2016) [44]
(Freeston et al., 1994 [94]; Buhr & Dugas, 2002 [95])			
Mizes Anorectic Cognitions Questionnaire-Revised	.90	-	Masuda et al. (2010) [53]
(Mizes et al., 2000) [96]			Masuda & Wendell (2010) [51]
Crandell Cognitions Inventory	.95	-	Gilbert & Christopher (2009) [33]
(Crandell & Chambless, 1986) [97]			
Emotion-Regulation Skills Questionnaire (subscale; Tolerance, Readiness to confront distressing situations)	.90 - .93	.75 - .78	Berking et al. (2012) [20]
(Berking & Znoj, 2008) [85]			
Rumination Reflection Questionnaire	.90 - .91	-	Coffey et al. (2010) [28]
(Trapnell & Campbell, 1999) [98]			
Cognitive Distortion Questionnaire	.85	.87	de Oliveira et al. (2015) [57]
(de Oliveira et al., 2015) [57]			
Automatic Thought Questionnaire-Negative	.98	-	Wong (2010) [69]
(Hollon & Kendall, 1980) [99]			
Automatic Thought Questionnaire-Positive	.97	-	Wong (2010) [69]
(Ingram & Wisnicki, 1988) [100]			
Scale of Ehinic Experience (subscale; Perceived discrimination)	.83 - .91	.77 - .86	Brittian et al. (2015) [25]
(Malcarne et al., 2006) [101]			Tucker et al. (2016) [65]
Acculturative Stress Scale for International Students (subscale; Perceived discrimination)	.92	-	Wong et al. (2014) [70]
(Sandhu & Asrabadi, 1994) [102]			
Everyday Discrimination Scale	.88	-	Chen et al. (2014) [27]
(Williams et al., 1997) [103]			
Ruminative Response Scale	.90	.67	Kneeland & Dovidio (2019) [42]
(Treynor et al., 2003) [104]			
White Bear Suppression Inventory	.89	.69	Kneeland & Dovidio (2019) [42]
(Wegner & Zanakos, 1994) [105]			Zawadzki et al. (2018) [72]
Thought Control Questionnaire	.67-.79	.67-.83	Zawadzki et al. (2018) [72]
(Wells & Davies, 1994) [106]			
Cognitive Fusion Questionnaire	.88-.93	.80	Krafft et al. (2019) [45]
(Gillanders et al., 2014) [107]			
**Behavior**			
Acceptance and Action Questionnaire	.88 - .90	.72	Masuda & Tully (2012) [50]
(Bond & Bunce, 2003) [108]			Masuda et al. (2009) [52]
			Masuda et al. (2010) [53]
			Woodruff et al. (2013) [71]
Dialectical Coping Scale	.81	-	Wang et al. (2016) [67]
(Wang et al., 2016) [67]			
Brief COPE Inventory	.81 - .88	-	Mahmoud et al. (2012) [48]
(Carver, 1997) [109]			
Emotional Intelligence Scale	.87	-	Birks et al. (2009) [22]
(Schutte et al., 1998) [110]			Jayalakshmi & Magdalin (2015) [39]
General Coping Questionnaire (subscale; dispositional coping)	.86 - .92	.63 - .86	Sasaki & Yamasaki (2005) [61]
(Sasaki & Yamasaki, 2002) [111]			
Religious Coping Scale	.97	-	Hovey & Seligman (2007) [37]
(Boudreaux et al., 1995) [112]			
Trait Meta-Mood Scale (subscale; Repair)	.86 - .90	-	Coffey et al. (2010) [28]
(Salovey et al., 1995) [83]			Costa et al. (2013) [29]
			Montes-Berges & Augusto (2007) [56]
Trait Coping Style Questionnaire	-	-	Luo & Wang (2009) [47]
(Wang, 1999) [113]			
Difficulties in Emotion Regulation Scale	.76 - .90	-	Coffey et al. (2010) [28]
(Gratz & Roemer, 2004) [114]			Mayorga et al. (2018) [54]
Proactive Coping Inventory	.80 - .85	-	Griva & Anagnostopoulos (2010) [34]
(Greenglass, 2002) [115]			
Brief Religious Coping Scale	.60 - .94	-	Khan et al. (2016) [40]
(Pargament et al., 2011) [116]			Kim et al. (2015) [41]
Cognitive Flexibility Scale	.72 - .82	-	Koesten et al. (2009) [43]
(Martin & Rubin, 1995) [117]			
Social Problem-Solving Inventory-Revised	.68 - .91	-	Ranjbar et al. (2013) [58]
(D' Zurilla et al., 2011) [118]			
Coping Orientation for Problem Experiences	.45 - .92	.46 - .86	Iwasaki (2003) [38]
(Carver et al., 1989) [119]			
Social Skills Inventory	.75-.88	.81-.96	Moeller & Seehuus (2019) [55]
(Riggio, 1986) [120]			
Coping Strategies Inventory	.71-.94	.67-.83	Sanchez et al. (2018a) [59]
(Tobin et al., 1989) [121]			Sanchez et al. (2018b) [60]
Confucian Coping Scale	.51-.77	-	Lihua et al. (2017) [46]
(Li & Hou, 2012) [122]			
Responses to Stress Questionnaire (subscale; engagement disengagement coping)	.80-.92	.69-.81	Bettis et al. (2017) [21]
(Connor-Smith et al., 2000) [123]			
Problem-Solving Questionnaire	.51-.86	-	Hipwell (2005) [36]
(Cassidy & Long, 1996) [124]			
**Negative affect**			
Positive and Negative Affect Schedule (subscale; Negative affect)	.84 - .87	.39 - .71	Anderson & Arnoult (1989) [19]
(PANAS; Watson et al., 1988) [125]			Deng et al. (2011) [30]
			Kraemer et al. (2016) [44]
			Wang et al. (2016) [67]
			Woodruff et al. (2013) [71]
			Mayorga et al. (2018) [54]
Depression Anxiety Stress Scale (subscale; Depression, Anxiety)	.90 - .95	-	Bowlin & Baer (2012) [24]
(DASS; Crawford, & Henry, 2003) [126]			Flett et al. (2016) [32]
			Hintz et al. (2015) [35]
			Mahmoud et al. (2012) [48]
			Vand et al. (2014) [66]
			Moeller & Seehuus (2019) [55]
Thought, Feeling, and Experience Questionnaire (subscale; Depression, Anxiety, Hopelessness)	.90	-	Thanoi & Klainin-Yobas (2015) [64]
(TEFQ; Thanoi et al., 2011) [127]			
Objectified Body Consciousness Scale (subscale; Body shame)	.72 - .89	.73 - .79	Calogero & Pina (2011) [26]
(McKinley & Hyde, 1996) [89]			
Body Image Guilt and Shame Scale (subscale; Body guilt)	.88 - .90	-	Calogero & Pina (2011) [26]
(Thompson et al., 2003) [128]			
Multiple Affect Adjective Check List	.72 - .85	.31 - .68	Anderson & Arnoult (1989) [19]
(Zuckerman, 1960) [129]			
**Positive affect**			
Positive and Negative Affect Schedule (subscale; Positive affect)	.86 - .90	.47 - .68	Deng et al. (2011) [30]
(PANAS; Watson et al., 1988) [125]			Wang et al. (2016) [67]
			Woodruff et al. (2013) [71]
Mental Health Inventory (subscale; positive affect)	.81 - .96	-	Sanchez et al. (2018b) [60]
(Veit & Ware, 1983) [130]			
**Happiness**			
Satisfaction with Life Scale	.87	.82	Costa et al. (2013) [29]
(SWLS; Diener et al., 1985) [131]			Wong (2010) [69]
			Woodruff et al. (2013) [71]
Fordyce Emotion Questionnaire	-	.86	Woodruff et al. (2013) [71]
(Fordyce, 1988) [132]			
Social and Emotional Health Surveys	.92	-	Marino et al. (2016) [49]
(SEHS; Furlong et al., 2014) [133]			
Spiritual Well-Being Scale (subscale; Existential well-being)	-	-	Disch et al. (2000) [31]
(Ellison, 1983) [134]			
Oxford Happiness Questionnaire-Short	.78	.86	Wong (2010) [69]
(Hills & Aygyle, 2002) [135]			
**Social function**			
Medical Outcomes Study Short Form Sruvey (subscale; Social functioning)	.80	-	Bodenlos et al. (2015) [23]b
(Ware & Sherbourne, 1992) [136]			
Interpersonal Relationship Harmony Inventory	-	-	Wang et al. (2016) [67]
(Kwan et al., 1997) [137]			
General Health Questionnaire (subscale; Social dysfunction)	.70 - .90	-	Koesten et al. (2009) [43]
(Goldberg, 1978) [138]			Ranjbar et al. (2013) [58]
			Sasaki & Yamasaki (2005) [61]
**Stress response**			
Interpersonal Reactivity Index (subscale; Personal distress)	.71 - .77	.62 - .71	Masuda & Wendell (2010) [51]
(Davis, 1983) [139]			Masuda et al. (2009) [52]
			Masuda et al. (2010) [53]
Perceived Stress Scale	.84 - .86	.85	Birks et al. (2009) [22]
(Cohen et al., 1983) [140]			Bodenlos et al. (2015) [23]
			Shapiro et al. (2011) [62]
			Kneeland & Dovidio (2019) [42]
			Bettis et al. (2017) [21]
Depression Anxiety Stress Scale (subscale; Stress)	.93	-	Bowlin & Baer (2012) [24]
(DASS; Crawford, & Henry, 2003) [126]			Hintz et al. (2015) [35]
			Mahmoud et al. (2012) [48]
Thought, Feeling, and Experience Questionnaire (subscale; Stress)	.90	-	Thanoi & Klainin-Yobas (2015) [64]
(TEFQ; Thanoi et al., 2011) [127]			
Counseling Center Assessment of Psychological Symptoms-34 (subscale; distress)	.76-.89	74-.87	Krafft et al. (2019) [45]
(Locke et al., 2012) [141]			
The Social, Attitudinal, Familial, and Environmental Scale	.89	-	Mayorga et al. (2018) [54]
(Mena et al., 1987)			
Responses to Stress Questionnaire (subscale; social stress)	.80-.92	.69-.81	Bettis et al. (2017) [21]
(Connor-Smith et al., 2000) [123]			
**Psychological symptoms**			
Beck Depression Inventory	.86	-	Anderson & Arnoult (1989) [19]
(Beck et al., 1961) [142]			Hovey & Seligman (2007) [37]
			de Oliveira et al. (2015) [57]
			Wong (2010) [69]
			Woodruff et al. (2013) [71]
			Kneeland & Dovidio (2019) [42]
			Lihua et al. (2017) [46]
Beck Anxiety Inventory	.92	.75	de Oliveira et al. (2015) [57]
(Beck et al., 1988) [143]			Wong (2010) [69]
			Woodruff et al. (2013) [71]
			Lihua et al. (2017) [46]
Center for Epidemiologic Studies Depression Scale	.85	.53	Brittian et al. (2015) [25]
(Radloff, 1977) [144]			Gilbert & Christopher (2009) [33]
			Tucker et al. (2016) [65]
Medical Outcomes Study Short Form Sruvey (subscale; Mental health 5)	.77	-	Montes-Berges & Augusto (2007) [56]
(Ware & Sherbourne, 1992) [136]			Sanchez et al. (2018a) [59]
Brief Symptom Inventory	.74 - .89	-	Berking et al. (2012) [20]
(Derogatis & Spencer, 1982) [145]			Coffey et al. (2010) [28]
			Masuda & Tully (2012) [50]
Hopelessness Depression Symptom Questionnaire	.93	-	Zhou et al. (2013) [73]
(Metalsky & Joiner, 1997) [146]			
Penn State Worry Questionnaire	.88	.79	Vand et al. (2014) [66]
(Meyer et al., 1990) [147]			
Eating Disorder Examination Questionnaire	.84 - .85	.81	Calogero & Pina (2011) [26]
(Mond et al., 2006) [148]			
Three Dichotomous Items	-	-	Su & Chen (2015) [63]
(Rost et al., 1993) [149]			
Posttraumatic Diagnostic Scale	.92	.83	Su & Chen (2015) [63]
(Foa et al., 1997) [150]			
Hopkins Symptom Checklist-21-item version	.90	-	Wong et al. (2014) [70]
(Green et al., 1988) [151]			
Patient Health Questionnaire-9 Scale	.89	-	Chen et al. (2014) [27]
(Kroenke et al., 2001) [152]			Bettis et al. (2017) [21]
Generalized Anxiety Disorder 7-item	.92	.83	Chen et al. (2014) [27]
(Spitzer et al., 2006) [153]			Bettis et al. (2017) [21]
General Health Questionnaire (subscale; Depression)	.70 - .90	-	Ranjbar et al. (2013) [58]
(Goldberg, 1978) [138]			Sasaki & Yamasaki (2005) [61]
SCL-90 Symptom checklist	.62 - .96	-	Luo & Wang (2009) [47]
(Derogatis, 1994) [154]			Wang et al. (2017) [68]
State-Trait Anxiety Inventory (subscale; Trait scale)	.92	-	Griva & Anagnostopoulos (2010) [34]
(Spielberger et al., 1970) [155]			
Personality Assessment Inventory (subscale; Anxiety)	.90	-	Hovey & Seligman (2007) [37]
(Morey, 1991) [156]			
Scale for Measuring Depression and Anxiety	.74	-	Khan et al. (2016) [40]
(Costello & Comrey, 1967) [157]			
PTSD Checklist	.97	.96	Zawadzki et al. (2018) [72]
(Weathers et al., 1993) [158]			
Inventory of Depression and Anxiety Symptoms	.77-.89	.72-.83	Mayorga et al. (2018) [54]
(Watson et al., 2007) [159]			
**Quality of life**			
World Health Organization Quality of Life-BREF	.68 - .82	-	Deng et al. (2011) [30]
(Skevington et al., 2004) [160]			Woodruff et al. (2013) [71]
**Well-being**			
Subjective Well-Being	.90	-	Shapiro et al. (2011) [62]
(Diener, 1984) [161]			
Scale of Psychological Well-Being	.86 - .93	.81 - .85	Bowlin & Baer (2012) [24]
(Ryff, 1989) [7]			Iwasaki (2003) [38]
Medical Outcomes Study Short Form Sruvey (subscale; Emotional well-being)	.80	-	Bodenlos et al. (2015) [23]
(Ware & Sherbourne, 1992) [136]			
Warwick-Edinburgh Mental Well-being Scale	.89 - .91	.83	Jayalakshmi & Magdalin (2015) [39]
(Tennant et al., 2007) [162]			
Mental Health Inventory (subscale; psychological well-being)	.81 - .96	-	Kim et al. (2015) [41]
(Veit & Ware, 1983) [130]			
**General Health**			
General Health Questionnaire	.70 - .90	-	Costa et al. (2013) [29]
(Goldberg, 1978) [138]			Koesten et al. (2009) [43]
			Masuda & Tully (2012) [50]
			Masuda & Wendell (2010) [51]
			Masuda et al. (2009) [52]
			Masuda et al. (2010) [53]
			Ranjbar et al. (2013) [58]
			Zawadzki et al. (2018) [72]
Mental Health Inventory	.81 - .96	-	Iwasaki (2003) [38]
(MHI; Veit and Ware, 1983) [130]			

^a^The psychometrics include measures of internal consistency (*α*) and test re-test reliability (*r*_xx_).

As Table 2 indicates, “behavior” (29 studies, 48 effect sizes) is the most common cognitive behavioral variable related to mental health status. The second most common is “thought” (23 studies, 33 effect sizes) and the third is “attention” (20 studies, 36 effect sizes). “Psychological symptom” (32 studies, 41 effect sizes) is the most common mental health category related to the cognitive behavioral variables. Psychological symptoms included “depressive symptoms,” “anxiety symptoms,”“pathological worry,” “post-traumatic stress disorder symptoms,” and the like (details are shown in Table 3). The second most common is “stress response” (14 studies, 17 effect sizes) and the third is “negative affect” (13 studies, 17 effect sizes). Table 4 shows the meta-analysis results for each classification.

**Table 4:** Estimated associations between cognitive behavioral variables and mental health.

Classification of mental health	Attention				Thought				Behavior			
	*r*	*N*^*a*^	Sample size	*I*^2^	*r*	*N*	Sample size	*I*^2^	*r*	*N*	Sample size	*I*^2^
	[95% CI]		(*n*)	(%)	[95% CI]		(*n*)	(%)	[95% CI]		(*n*)	(%)
Negative affect	-.39	6	5,275	84.5	.46	7	9,696	94.7	-.40	4	1,873	95.1
	[-.47, -.31]				[.35, .58]				[-.63, -.17]			
Positive affect	.23	2	557	0.0	-	0	-	-	.21	3	1,093	94.8
	[.15, .31]								[-.07, .49]			
Happiness	.28	4	19,111	96.9	-	1	1,592	-	.39	3	1,634	88.7
	[.15, .41]								[.23, .56]			
Social function	-	1	1,860	-	-	0	-	-	.19	4	5,628	94.5
									[.16, .46]			
Stress response	-.35	5	3,684	80.9	.54	6	3,463	96.7	-.47	6	2,293	73.1
	[-.45, -.25]				[.31, .77]				[-.55, -.39]			
Psychological symptom	-.32	9	7,883	96.6	.43	15	8,615	95.8	-.29	17	18,041	97.7
	[-.46, -.18]				[.32, .54]				[-.40, -.19]			
Quality of life	.32	2	557	0.0	-	0	-	-	-	1	147	-
	[.23, .40]											
Well-being	.39	3	3,292	69.5	-	0	-	-	.23	3	1,189	0.1
	[.31, .47]								[.17, .29]			
General health	.32	4	3,042	90.9	-.36	4	2,035	0.0	.38	7	6,128	91.3
	[.17, .48]				[-.40, -.32]				[.29, .47]			
Overall	.34	36	45,261	91.8	.46	33	26,802	96.5	.33	48	37,901	96.0
	[.30, .38]				[.39, .53]				[.27, .38]			
	.32^a^											
	[.28, .36]^b^											

^a^*N* = number of studies

^b^Based on Trim and Fill method

### Relationship between attention and mental health status

**Fig 2:**
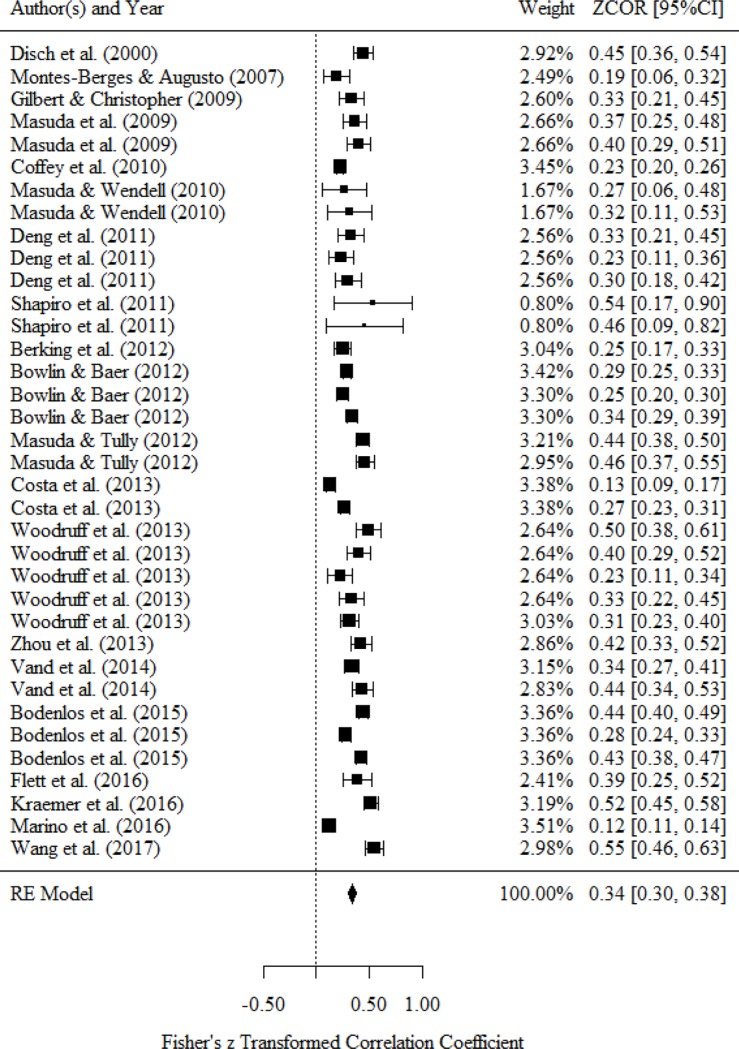
Forest plot of the relationship between attention and mental health.

Studies on attention measured the awareness of personal experiences such as body sensation, thought, and emotion (e.g., mindfulness, metacognitive awareness). Table 4 and Fig 2 present a medium correlation between attention and mental health status (*r* = .34, 95% confidence interval [CI] = .30 to .38). Because we observed a large statistical heterogeneity (*I*^*2*^ = 91.8%), we conducted a subgroup analysis based on the classification of mental health status.

Six studies reported a bivariate relationship between attention and negative affect. The results of this meta-analysis indicate a medium correlation between attention and negative affect (*r* = -.39, 95% CI = −.47 to −.31). Two studies reported a bivariate relationship between attention and positive affect. The results of this meta-analysis indicate a small or medium correlation between attention and positive affect (*r* = .23, 95% CI = .15 to .31). Four studies reported a bivariate relationship between attention and happiness. The results of this meta-analysis indicate a small or medium correlation between attention and happiness (*r* = .28, 95% CI = .15 to .41). Five studies reported a bivariate relationship between attention and stress response. The results of this meta-analysis indicate a medium correlation between attention and stress response (*r* = −.35, 95% CI = −.45 to −.25). Nine studies reported a bivariate relationship between attention and psychological symptom. The results of this meta-analysis indicate a medium correlation between attention and psychological symptom (*r* = −.32, 95% CI = −.46 to −.18). Two studies reported a bivariate relationship between attention and QOL. The results of this meta-analysis indicate a medium correlation between attention and QOL (*r* = .32, 95% CI = .23 to .40). Three studies reported a bivariate relationship between attention and well-being. The results of this meta-analysis indicate a medium correlation between attention and well-being (*r* = .39, 95% CI = .31 to .47). Four studies reported a bivariate relationship between attention and general health. The results of this meta-analysis indicate a medium correlation between attention and general health (*r* = .32, 95% CI = .17 to .48). We did not conduct subgroup analysis because only one study reported a bivariate relationship between attention and social function.

### Relationship between thought and mental health status

**Fig 3:**
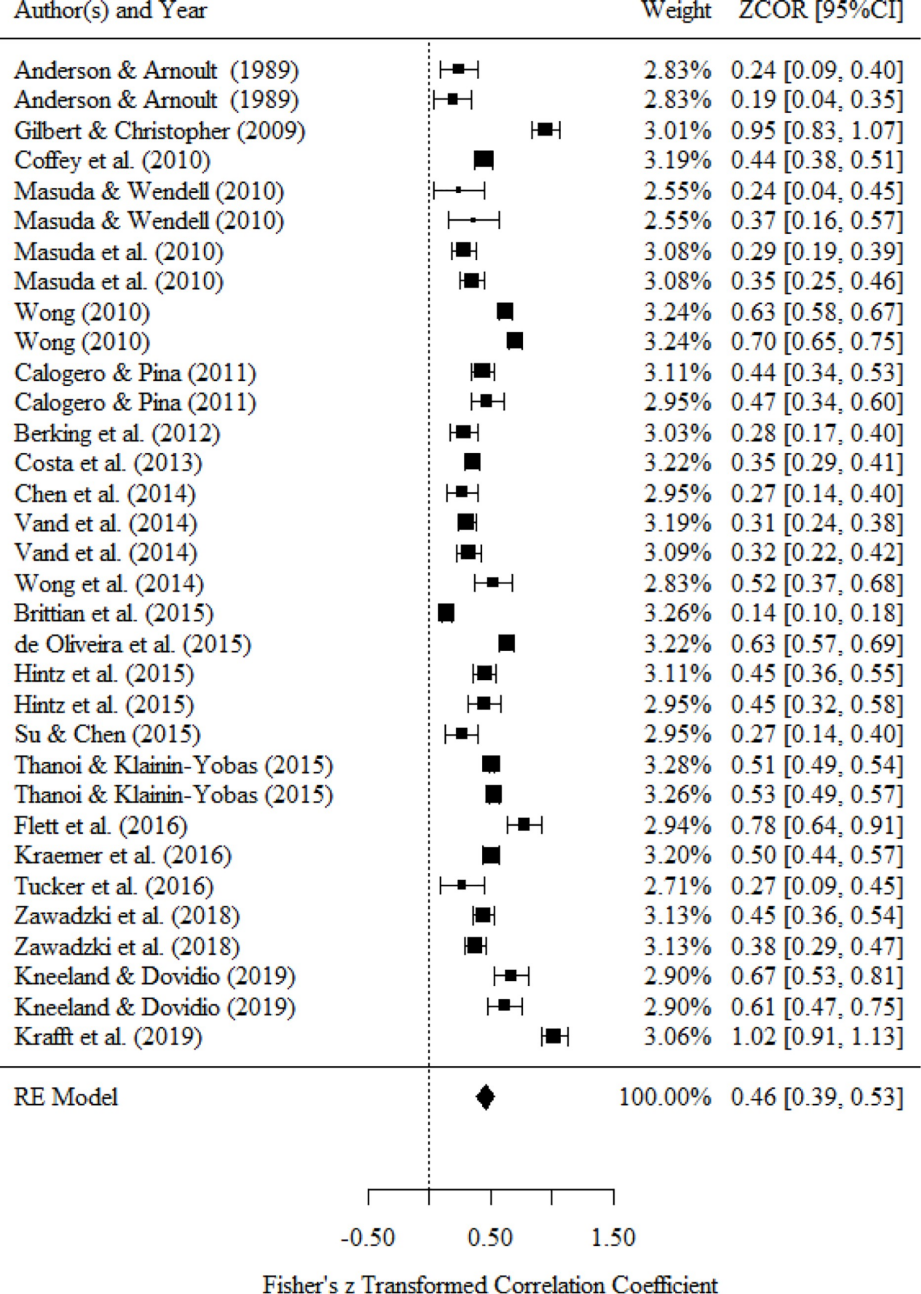
Forest plot of the relationship between thought and mental health.

Studies on thought measured thinking variables (e.g., automatic thoughts, irrational belief). Table 4 and Fig 3 present a medium or large correlation between thought and mental health status (*r* = .46, 95% CI = .39 to .53). Because we observed a large statistical heterogeneity (*I*^*2*^ = 96.5%), we conducted a subgroup analysis based on the classification of mental health status.

Seven studies reported a bivariate relationship between thought and negative affect. As a result of integrating the effect size, we found a medium or large correlation between thought and negative affect (*r* = .46, 95% CI = .35 to .58). Six studies reported a bivariate relationship between thought and stress response. As a result of integrating the effect size, we found a large correlation between thought and stress response (*r* = .54, 95% CI = .31 to .77). Fifteen studies reported a bivariate relationship between thought and psychological symptom. As a result of integrating the effect size, we found a medium or large correlation between thought and psychological symptom (*r* = .43, 95% CI = .32 to .54). Four studies reported a bivariate relationship between thought and general health. As a result of integrating the effect size, we found a medium correlation between thought and general health (*r* = −.36, 95% CI = −.40 to −.32). We did not conduct subgroup analysis because no studies or only one study reported a bivariate relationship between positive affect, happiness, social function, QOL, and well-being.

### Relationship between behavior and mental health status

**Fig 4:**
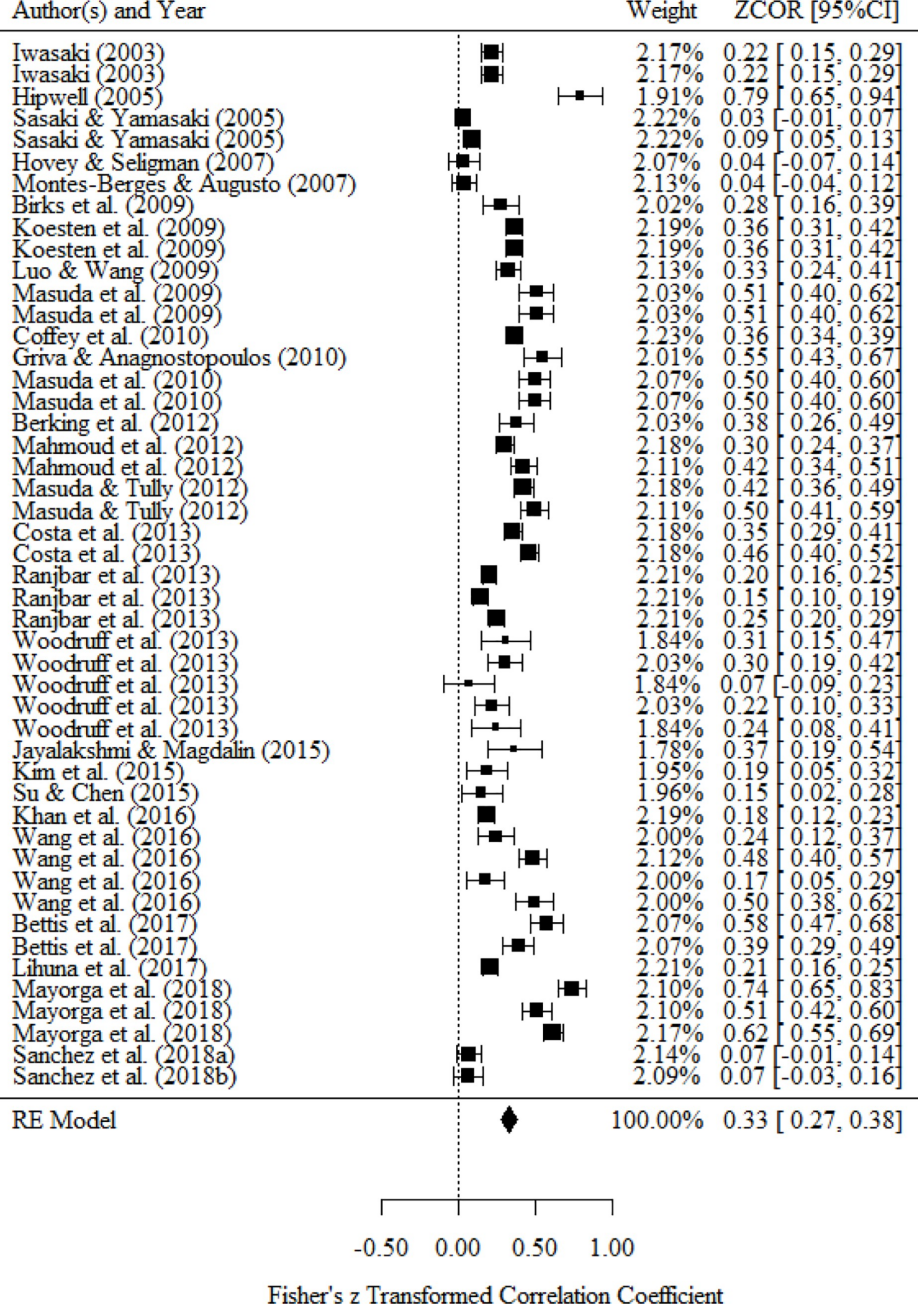
Forest plot of the relationship between behavior and mental health.

Studies on behavior measured coping processes of external or internal experiences (e.g., problem-solving coping, commitment). Table 4 and Fig 4 presents a medium correlation between behavior and mental health status (*r* = .33, 95% CI = .27 to .38). Because a large statistical heterogeneity was observed (*I*^*2*^ = 95.2%), we conducted a subgroup analysis based on the classification of mental health status.

Four studies reported a bivariate relationship between behavior and negative affect. As a result of integrating the effect size, we found a small or medium correlation between thought and negative affect (*r* = −.40, 95% CI = −.63 to −.17). Three studies reported a bivariate relationship between behavior and positive affect. There is no significant correlation between behavior and positive affect as a result of integrating the effect size (*r* = .21, 95% CI = −.07 to .49). Three studies reported a bivariate relationship between behavior and happiness. As a result of integrating the effect size, we found a medium correlation between behavior and happiness (*r* = .39, 95% CI = .23 to .56). Four studies reported a bivariate relationship between behavior and social function. As a result of integrating the effect size, we found a small correlation between behavior and social function (*r* = .19, 95% CI = .07 to .31). Six studies reported a bivariate relationship between behavior and stress response. As a result of integrating the effect size, we found a medium or large correlation between behavior and stress response (*r* = −.47, 95% CI = −.55 to −.39). Seventeen studies reported a bivariate relationship between behavior and psychological symptom. As a result of integrating the effect size, we found a medium correlation between behavior and psychological symptom (*r* = −.29, 95% CI = −.40 to −.19). Three studies reported a bivariate relationship between behavior and well-being. As a result of integrating the effect size, we found a small or medium correlation between behavior and well-being (*r* = .23, 95% CI = .17 to .29). Seven studies reported a bivariate relationship between behavior and general health. As a result of integrating the effect size, we found a medium correlation between behavior and general health (*r* = .38, 95% CI = .29 to .47). We did not conduct a subgroup analysis because only one study reported a bivariate relationship between behavior and QOL.

### Reporting bias

**Fig 5:**
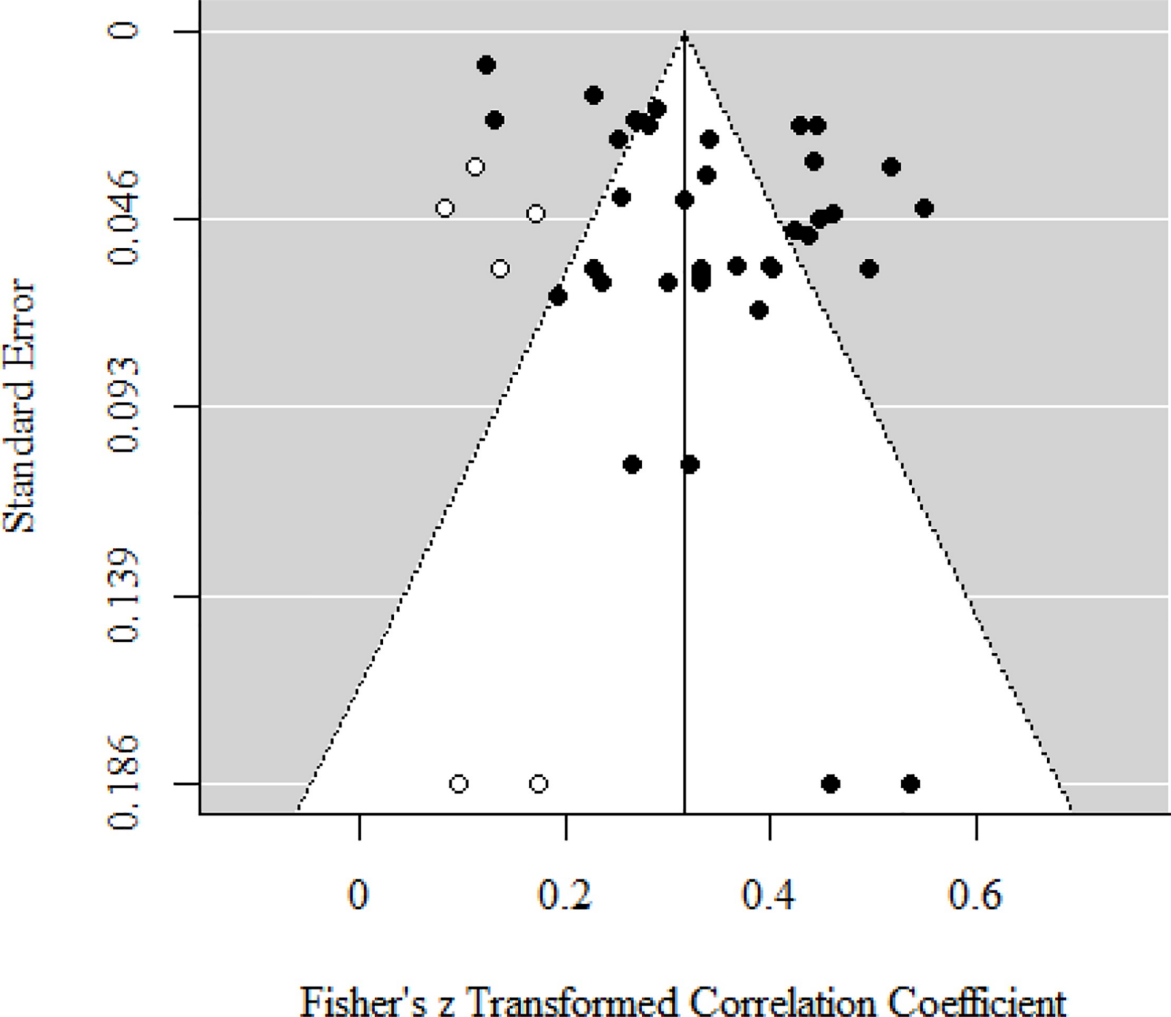
Funnel plot of the relationship between attention and mental health. Black circles: included studies, White circles: added possible missing studies using Trim and Fill methods.

We assessed the risk of reporting bias through visual inspection and linear regression tests of funnel plots [77]. Because it has been argued that the test for funnel plot asymmetry should be used only when there are at least 10 studies [18], we only conducted a linear regression test when there were over 10 studies. With the linear regression test, asymmetry of the funnel plot was detected in studies that reported bivariate relations between attention and mental health status (*p* < .001). Based on the trim and fill method, the uncorrected effect size (before adding the possible missing studies) is .34 (95% CI = .30 to .38), and the corrected effect size (after adding six possible missing studies) is .32 (95% CI = .28 to .36). Although there is evidence of publication bias, its effect is not significant (Fig 5).

## Discussion

This study is the first meta-analysis of the relationship between cognitive behavior variables and mental health status in university students. We found that the overall correlation coefficients between cognitive behavioral variables and mental health status were medium (attention: *r* = .32; thought: *r* = .46; behavior: *r* = .33). However, we detected a large heterogeneity (attention: *I*^*2*^ = 91.8%; thought: *I*^*2*^ = 96.5%; behavior: *I*^*2*^ = 96.0%), which means that the effect sizes likely depend on mental health status, while cognitive behavioral variables are related to mental health status. Therefore, we classified mental health as “negative affect,” “positive affect,” “happiness,” “social function,” “stress response,” “psychological symptom,” “QOL,” “well-being,” and “general health” and conducted a meta-analysis on them.

As Table 4 shows, attention has larger effect sizes than behavior in relation to well-being. For positive affect and QOL, attention shows significant effect sizes, but thought and behavior do not. Thought has larger effect sizes than attention and behavior on negative affect, stress response, and psychological symptom. In social function, behavior shows significant effect sizes, but attention and thought do not. These findings suggest that attention is related to the positive aspects of mental health such as well-being, and thought is related to the negative aspects of mental health such as negative affect. Behavior is related to social function, but attention and thought are not.

As mentioned above, this study identifies cognitive behavioral variables that are strongly related to the mental health status in university students. Next, we review how these cognitive behavioral variables have been used in existing psychotherapy. To develop effective psychological intervention methods, we will consider what kind of future research is necessary.

These results support previous studies that examined the effects of psychological treatment. First, the classification of attention in this study includes mindfulness and metacognitive awareness (e.g., Mindful Attention Awareness Scale [81], Metacognition Questionnaire [84]). As techniques to promote mindfulness and metacognitive awareness, mindfulness-based psychotherapy [163], attention training [164], and computer-based training to train attention [165] are available [12]. Mindfulness-based psychotherapy is the most frequently reported and effective technique by clinical trials and meta-analyses [166–168]. Mindfulness is defined as “paying attention in a particular way: on purpose, in the present moment, and nonjudgmentally” [169]. Several clinical trials and meta-analyses report that mindfulness-based psychotherapy is effective when it creates positive aspects in mental health [170,171]. Furthermore, integrating 23 meta-analyses that reported on the effectiveness of mindfulness-based psychotherapy revealed that mindfulness-based psychotherapy not only improved depressive symptoms (standard mean difference (SMD) = −.37) and anxiety symptoms (SMD = −.48) but also promoted QOL (SMD = −.39) [172]. In previous research, while the subjects were not purely university students, mindfulness-based psychotherapy not only improved the negative aspects of mental health but also promoted the positive aspects. In addition, in the present study, the attention process was correlated with positive aspects of mental health in university students [25,26,28], suggesting that psychological interventions targeting the attention process may be effective when promoting positive mental health in this population.

Second, the classification of thought in this study includes automatic thoughts and dysfunctional belief (e.g., Automatic Thought Questionnaire-Negative [99], Dysfunctional Belief and Attitudes about Sleep Scale [90]). Cognitive therapy is a technique to improve automatic thoughts and dysfunctional belief [12]. Cognitive therapy is a psychological treatment focused on thought that improves depressive symptoms and supports clients by observing and considering the thought processes [173]. The Society of Clinical Psychology reported that cognitive therapy is an effective treatment for depression [174]. A meta-analysis suggests that the cognitive therapy treatment of depression has a higher remission rate as opposed to no intervention (Odds Ratio = 0.42) [175]. Another meta-analysis shows that cognitive therapy improves generalized anxiety and social anxiety [176,177]. In previous research, while subjects were not purely university students, cognitive therapy improved the negative aspects of mental health. In the present study, the thought process was correlated with the negative aspects of mental health in university students [33,57]; so psychological interventions targeting the thought process may be effective treatments for the negative aspects of mental health in this population.

Third, the classification of behavior in this study included coping and commitment (e.g., Brief COPE Inventory [109], Acceptance and Action Questionnaire [108]). As techniques to promote coping and commitment, behavioral activation and acceptance and commitment therapy are available [12]. Behavioral activation is a psychological treatment that focuses on increased engagement in adaptive activities, decreased engagement in activities that maintain depression or increased risk of depression, and solving problems that limit access to rewards or that maintain or increase aversive control [178]. Acceptance and commitment therapy is a psychological treatment that focuses on decreasing experiential avoidance and increasing action along the valued direction [179]. Behavioral activation and acceptance and commitment therapy are effective in improving social dysfunctions because they aim to resolve problems by focusing on real-life behavior. In randomized controlled trials, behavioral activation and acceptance and commitment therapy are shown to be effective against social dysfunction (behavioral activation: *d* = 1.21 [180]; acceptance and commitment therapy: *partial η*^2^ = .22 [181]). In previous research, while subjects were not purely university students, behavior activation and acceptance and commitment therapy improved social dysfunction. In the present study, the behavior process was correlated with the social function in university students [43,58], therefore, psychological interventions that target the behavior process may be effective when it comes to social dysfunction in university students. As mentioned above, when providing psychological interventions to university students, it would be best to provide psychotherapy that focuses on the attention, thought, and behavior variables that target mental health problems.

This meta-analysis is not without limitations. First, we detected a large heterogeneity in the studies included in the meta-analysis. The heterogeneity did not affect the results of the present study because this meta-analysis used the random effect model. However, future studies must consider heterogeneities among university students. Studies focusing on university students have at times taken into consideration several demographic variables, such as a student’s major [182]. In contrast, some studies were conducted without considering the differences in demographics [183]. These differences in demographics may affect the results of the analysis [22]. In addition, because the present study extracted only English articles, which is an international language, the influence of the cultural background could not be verified. In the future, it is necessary to analyze the data pertaining to each demographic, including cultural background, and accumulate the findings. Furthermore, we could not conduct a meta-analysis on some of the classifications because we could not extract the required amount of data. Therefore, some relationships between cognitive behavioral variables and mental health status were unclear (e.g., thought and positive aspects of mental health [positive affect, happiness, QOL, and well-being]); it will be necessary to try and resolve this issue in the future.

## Conclusion

The present study is the first to examine the relationship between cognitive behavioral variables and mental health status among university students using meta-analysis. The findings reveal that cognitive behavioral variables are overall correlated with mental health status. Therefore, psychological treatment based on CBT is effective for solving mental health problems among university students. Psychological treatment, including thought process, can be effective in treating the negative aspects of mental health, and the attention process can be effective in treating the positive aspects of mental health. However, this meta-analysis could not reveal some of the relationships between cognitive behavioral variables and mental health status.

In summary, psychological treatment based on CBT is effective in solving mental health problems among university students. However, outcomes vary, and several factors influence them. Therefore, when examining the effects of psychological treatment on university students, various outcomes should be included.

## Supporting information

S1 Table PRISMA checklist. (DOC)
